# Targeted interneuron ablation in an mTORopathy model: Testing a two-hit mechanism of epileptogenesis

**DOI:** 10.1016/j.pneurobio.2026.102925

**Published:** 2026-05-05

**Authors:** Austin W. Drake, Mary R. Dusing, Candi L. LaSarge, Carlie McCoy, Lilian Jerow, Sanskriti Vaghela, Justin V. Ruksenas, Steve C. Danzer

**Affiliations:** a Department of Anesthesia, Cincinnati Children’s Hospital Medical Center, Cincinnati, OH, United States; b Neuroscience Graduate Program, University of Cincinnati College of Medicine, Cincinnati, OH, United States; c Medical Scientist Training Program, University of Cincinnati College of Medicine, Cincinnati, OH, United States; d Department of Anesthesiology, University of Cincinnati College of Medicine, Cincinnati, OH, United States

**Keywords:** Pten, Somatostatin, Parvalbumin, Dentate gyrus, Focal cortical dysplasia, Diphtheria toxin

## Abstract

**Objective:**

Somatic mutations in mechanistic target of rapamycin (mTOR) pathway genes produce focal brain malformations that can lead to epilepsy. Malformations show a high degree of mosaicism, sometimes with less than 1% of neurons carrying the mutation. Seizures are hypothesized to be driven by mutation-carrying dysmorphic excitatory neurons, but lesions in patients and animal models also show loss of interneurons. Here, we queried whether interneuron loss could act as a second hit, releasing the brake on excitatory dysmorphic neurons and increasing epilepsy severity.

**Methods:**

To test this hypothesis, we developed a two-hit mouse model in which we combined loss of the mTOR pathway inhibitor phosphatase and tensin homologue (Pten) from excitatory hippocampal dentate granule cells with ablation of local parvalbumin or somatostatin interneurons. Pten was deleted from roughly 3% of granule cells, a level which is subthreshold for producing frequent generalized seizures, thus facilitating assessment for synergistic effects.

**Results:**

Pten loss alone produced occasional seizures, while interneuron ablation alone initiated frequent seizures lasting for about one week, followed by a significant decline in seizure incidence in subsequent weeks. The combination of Pten deletion and interneuron ablation did not produce a synergistic increase in seizure incidence over ablation alone.

**Interpretation:**

Findings confirm the potential for interneuron loss to drive epileptogenesis, but do not support the hypothesis that rapid disinhibition of Pten knockout granule cells enhances their ictogenic potential. Results provide new insights into the role of GABAergic inhibitory input in regulating the activity of mTOR hyperactive neurons.

## 1. Introduction

Mutations affecting more than a dozen genes in the mechanistic target of rapamycin (mTOR) pathway are a common cause of epilepsy in children (Wong et al., 2024). These mTORopathies encompass numerous syndromes, including hemimegalencephaly, tuberous sclerosis complex, and focal cortical dysplasia type II (FCDII). Disease is hypothesized to be driven by somatic mTOR-hyperactivating mutations in neural progenitor cells, producing mosaic malformations containing mixed populations of wildtype and mutation-carrying cells (Chung et al.,2023; Baldassari et al., 2019, 2024; Marsan and Baulac, 2018). Although variant allele frequency (VAF) reaches double digits in affected tissue for many patients, epileptogenic malformations can have VAFs of less than 1% (Carton et al., 2024; Kim et al., 2023; Krochmalnek et al., 2023). While mTOR pathway mutations are a well-established cause of cortical epilepsy, recent studies have also identified temporal lobe epilepsy patients with FCDII (Tran et al., 2025; Mao et al., 2019) and somatic mTOR mutations with similar low VAFs (Carton et al., 2024). A key unresolved question in the field, therefore, is how focal lesions with low VAFs produce severe neurological deficits like seizures.

Excitatory neurons with mTOR mutations undergo a dramatic transformation. Somas triple in size, dendritic fields expand, axon structure is altered, and excitability can be increased (Kwon et al., 2003; Arafa et al., 2019; Nguyen et al., 2024). Such dysmorphic neurons are readily evident in animal models and patient tissues (also known as cytomegalic or giant cells). The dramatic changes have led investigators to propose the mTOR mutant neurons may act as “hub cells”, with extensive inputs and outputs. In computational modeling studies, hub cells can drive epileptogenesis (Bui et al., 2015; Hadjiabadi et al., 2021). Interneurons could play a critical role in constraining the activity of hub cells and, intriguingly, dysmorphic neurons have been described in patient tissues which are encased in parvalbumin immunoreactive puncta (Szekeres-Paraczky et al., 2022; Spreafico et al., 1998; André et al., 2010). It is notable, therefore, that mTORopathies are associated with interneuron loss in humans (Nakagawa et al., 2017; Thom et al., 2003; Cheng et al., 2022; Zamecnik et al., 2006) and animal models (Yang et al., 2022; LaSarge et al., 2021; Drake et al., 2024; Zhong et al., 2021).

For the present study, we tested a two-hit model of epileptogenesis in an mTORopathy model. The model follows a hypothesized mechanism in which somatic mutations in excitatory progenitor cells occurring during brain development (1st hit) are followed by secondary interneuron loss in adulthood (2nd hit). Disinhibition of mTOR mutant neurons following interneuron loss is predicted to lead to a dramatic increase in seizure severity. To test our model, we combined conditional, inducible transgenic mice, to delete the mTOR negative regulator Pten from about 3% of hippocampal dentate granule cells (DGC), with a transgenic/viral strategy, to ablate about 50% of parvalbumin (PV) or somatostatin (SST) interneurons from the dentate gyrus. Previous studies in DGC-Pten knockout (KO) mice indicate that 3–5% granule cell deletion rates can drive local circuit changes and focal seizures, but not generalized seizures (LaSarge et al., 2021; Pun et al., 2012). The hippocampal model takes advantage of the well-characterized interneuron circuitry of the dentate gyrus, in which PV and SST interneurons in the dentate mediate feedforward and feedback inhibitory control of granule cells (Houser, 2007). Specifically, PV interneurons provide perisomatic inhibition, which leaves them positioned to limit seizure generation or propagation (Freund and Katona, 2007). SST interneurons, on the other hand, synapse onto distal dendrites, which enables them to exert influence by regulating input to granule cells (Peng et al., 2013; Leranth et al., 1990). Given how these interneuron populations exert influence over granule cell activity, their loss was predicted to drive DGC-Pten KO mice from a mild to severe seizure phenotype.

## 2. Materials and methods

### 2.1. Animals

Studies were approved by CCHMC’s Institutional Animal Care and Use Committee. Details on transgenic mouse lines are provided in Supplemental Table 1. Study animals were generated using Gli1-CreER^T2^ (Gli1^tm3(cre/ERT2)Alj^/J), Pten^fl/fl^ (*Pten*^tm1Hwu^/J), PV-FlpO (*Pvalb*^tm4.1(flpo)Hze^/J), SST-FlpO (*Sst*^*t*m3.1(flpo)Zjh^/J) and tdTomato reporter (tdT+; Gt (ROSA)26Sor^tm14(CAG-tdTomato)Hze^/J) mouse lines obtained from the Jackson Laboratory, all on a C57BL/6 background. Animals were housed together (up to 4 mice per cage) with a 14:10 h light/dark cycle.

For immunohistochemical analyses of inhibitory synapses, Gli1-CreER^T2^, Pten^fl/fl^, tdT+ (DGC-Pten KO; n = 6, 3 F/3 M) and Gli1-CreER^T2^, Pten^wt/wt^, tdT+ (control; n = 9, 6 F/3 M) mice were generated. All mice were treated with tamoxifen (250 mg/kg s.c., dissolved in corn oil at a concentration of 20 mg/mL) on postnatal day 14 (p14) to induce recombination in greater than 10–20% of granule cells (high DGC-Pten KOs), a threshold sufficient to cause spontaneous seizures (Pun et al., 2012).

To examine the impact of parvalbumin (PV) or somatostatin (SST) interneuron ablation in DGC-Pten KOs, triple transgenic Gli1-CreER^T2^, Pten^fl/fl^, PV-FlpO and Gli1-CreER^T2^, Pten^fl/fl^, SST-FlpO mice were generated (Table 1). All mice were treated with tamoxifen (250 mg/kg s. c.) on postnatal day 21 (p21) to induce recombination in fewer than 10% of granule cells (Low DGC-Pten KOs), below the threshold for generalized seizures (LaSarge et al., 2021).

#### 2.1.1. Surgeries

Mice were prepared for infusion of AAV9-CAG-frt-DTr (Vector Builders, VB190421–1029dxb; pAAV[Exp]-CAG>FRT1-F5-rev(DTR)-rev(FRT1)-rev(F5):WPRE) and EEG implantation as described previously (McCoy et al., 2025; Dusing et al., 2024 Apr). To target dorsal and ventral dentate gyrus bilaterally, AAV was infused at 4 sites (bregma, −2.3 mm, medial/lateral ±1.3 mm, depth 2.5 mm; bregma, −3.0 mm, medial/lateral ±2.75 mm, depth 2.75 mm). 500 nL was infused per site at 100 nL/min (2 × 10^8^ viral genomes per animal). Immediately following infusion, mice were implanted with cortical electrodes (Data Sciences International, ETA-F10) with leads placed over dura using the two anterior holes (Rowley et al., 2017). After recovery, mice were individually housed with food and water *ad libitum*. Animals were randomly assigned to receive daily intraperitoneal injections of either diphtheria toxin (DT; 30 μg/kg) or saline for 5 days. In prior studies, no effect of DT treatment on EEG metrics was observed in animals not expressing the diphtheria toxin receptor (Dusing et al., 2024 Apr; Hosford et al., 2016, 2017).

#### 2.1.2. EEG analysis

Band pass filtered (1–70 Hz) EEG data was scored using NeuroScore software (Data Sciences International). Seizures were defined as events with a > 2x increase in amplitude, duration > 10 s, and evolution of spike shape or frequency. Persistent epileptiform activity was defined as continuous spiking with seizures occurring at intervals < 5 min and no return to baseline EEG between seizures. For seizure frequency quantification, these periods were scored as a single event. Seizure frequency was determined for the entire 24/7 video-EEG recording period for each animal. Interictal spikes were quantified for the full 24-hour period immediately prior to the first DT or saline injection (baseline), followed by 24-hour assessments beginning the first and second weeks after treatment. Automated interictal spike detection parameters were optimized for each animal to ensure high sensitivity and were manually reviewed to remove artifacts.

#### 2.1.3. Immunohistochemistry

Brains were collected for histological studies using established protocols (Drake et al., 2024). Details on primary and secondary antibodies are provided in Supplemental Table 2. For immunostaining, slide-mounted tissue was permeabilized overnight in a solution consisting of 3% Triton-100, 0.75% glycine in 0.1 M phosphate-buffered saline (PBS) and then blocked in 5% normal goat or donkey serum, 0.75% glycine, and 1.5% Triton-100 in PBS for at least one hour. Slides were incubated overnight in primary antibodies in blocking solution, followed by a 4-hour incubation in secondary antibodies.

#### 2.1.4. Super resolution imaging of gephyrin and parvalbumin puncta

PV and gephyrin immunostaining were imaged on a Nikon Ti2 Microscope with an NSPARC detector and 100x oil objective (NA 1.45, 0.125 μm step, resolution 0.07 μm/pixel). For each animal, regions of interest (ROIs) were drawn for the inner (adjacent to the hilus) and outer (adjacent to the molecular layer) halves of the dentate granule cell body layer. Imaris software was used to count all puncta within each ROI and calculate puncta density per μm (Baldassari et al., 2019). Within each ROI, Imaris software was used to reconstruct granule cell soma surfaces and determine the number of PV and gephyrin puncta within 0.2 μm of the soma surface. Soma surface area was used to calculate puncta density per cell in μm^2^. For each animal, measures from 5 to 10 cells were averaged for analysis.

#### 2.1.5. Quantification of Pten deletion rates and interneuron density

The percentage of dentate granule cells lacking Pten was determined from brain sections (bregma −2.00 to −2.30) immunostained for Pten and counterstained with Nuclear Blue using established protocols (Arafa et al., 2019). To examine diphtheria toxin receptor (DTr) labeling of SST or PV interneuron populations and quantify interneuron loss, confocal images of the dentate hilus were collected from left and right hippocampi of sections from dorsal (bregma −1.6 mm) and ventral (bregma −3.3 mm) hippocampus using a 20x water objective (NA 0.95, 4 μm step, resolution 1.24 μm/pixel). SST and PV interneuron numbers were quantified from the four dentate hilar regions per mouse using Nikon software (PV) or spot detector with Imaris software (SST) and converted to density per hilar area (cells/1mm^3^).

#### 2.1.6. Analysis of somatostatin immunostaining in the dentate molecular layer

Single confocal optical sections of SST-immunostaining in the dentate molecular layer were captured with a 60x water objective (NA 1.27, resolution 0.21 μm/pixel). Optical sections were analyzed in Nikon Elements AR 5.20.21 64-bit. The inner molecular layer was defined as the 17% closest to the granule cell body layer, while the middle and outer layers were an equal split of the remainder (Santos et al., 2011). To calculate the area occupied by SST-immunoreactive fibers in each layer, a binary was created using Nikon’s auto threshold feature, creating an overlay of the SST immunostaining.

#### 2.1.7. Statistical analysis and figure preparation

Investigators were blind to animal treatment and genotype for all data collection and analysis. Male and female mice were binned for analyses. SigmaPlot (Version 15.0) and GraphPad Prism (Version 10) were used for statistical analyses. Representative images were processed in NIS-Elements AR (Version 5.42.03) and figures were created using GraphPad Prism and Adobe Photoshop CS5.

## 3. Results

### 3.1. Pten knockout granule cells are surrounded by PV- and gephyrin-immunoreactive puncta

**Fig. 1.**
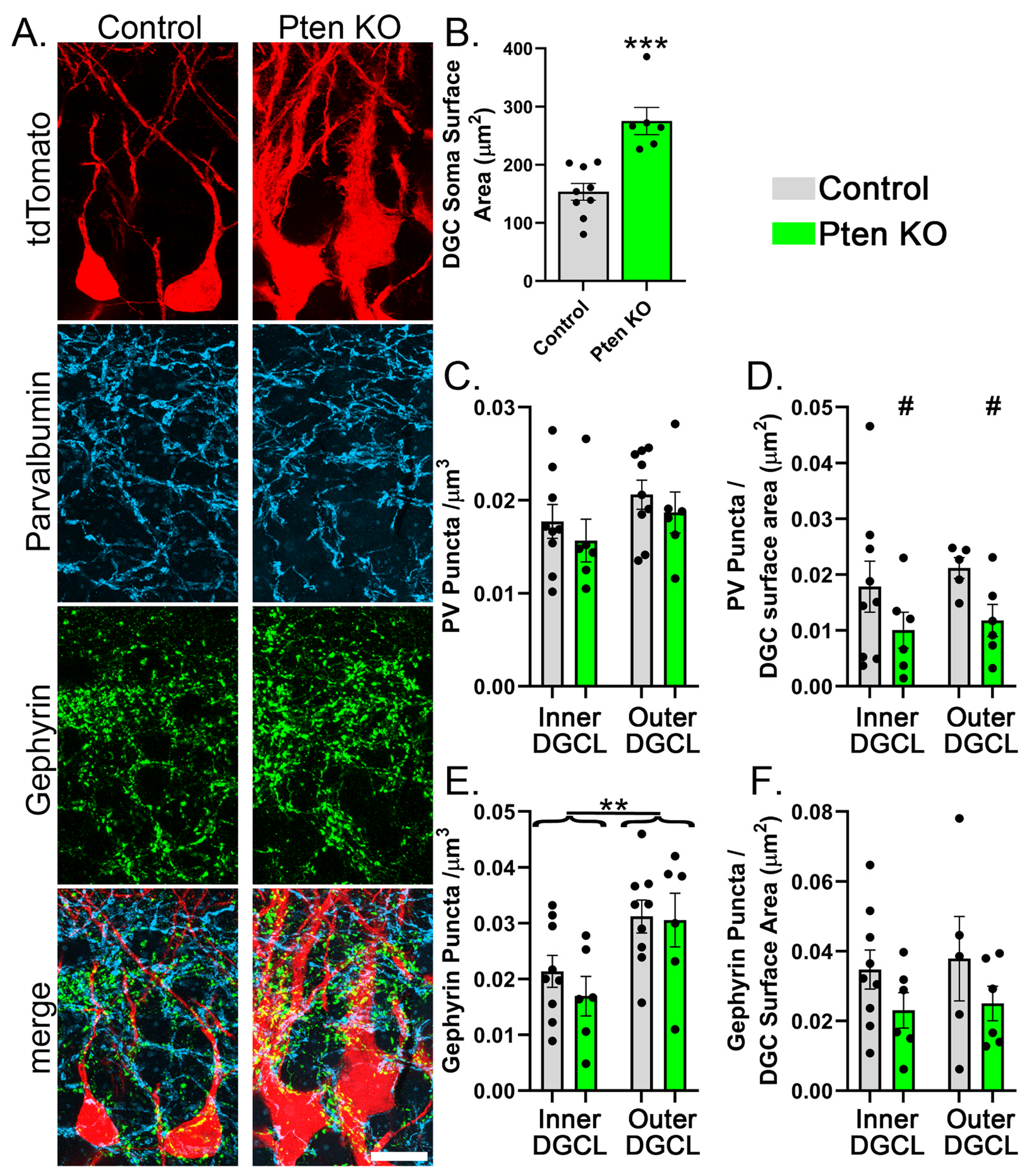
**A)** Confocal images showing tdTomato reporter-labeled hippocampal granule cells (red) from a control mouse and a Pten KO mouse. Note the enlarged somas of Pten KO granule cells. Immunostaining for PV (blue) and the postsynaptic inhibitory marker gephyrin (green) show abundant puncta around the somas and proximal dendrites of both control and Pten KO granule cells. Scale = 10 μm. **B)** Soma surface area was significantly increased for Pten KO relative to control dentate granule cells. **C)** The density of PV puncta was statistically similar in the inner and outer regions of the granule cell body layer. **D)** The density of PV puncta apposed to granule cell somas was significantly reduced for Pten KO cells relative to control cells. #, p < 0.05, main effect of genotype. **E)** The density of gephyrin puncta was significantly greater in the outer half of the cell body layer relative to the inner half. **, p < 0.01, main effect of region **F)** The density of gephyrin puncta apposed to granule cell somas did not differ significantly between Pten KO and control cells.

To establish whether Pten KO cells receive robust inhibitory input, the density of presynaptic PV-immunoreactive and postsynaptic gephyrin-immunoreactive puncta was quantified in control mice and mice with Pten deleted from greater than 10% of granule cells (High DGC-Pten KO). Granule cells labeled with tdTomato from both control and DGC-Pten KO mice were densely decorated with PV and gephyrin puncta, indicative of robust inhibitory input (Fig. 1A). Consistent with previous studies (LaSarge et al., 2019), soma surface area was significantly increased for Pten KO relative to control cells (Fig. 1B; *t*-test, n = 9 control and 6 Pten KO mice, 5–10 cells/animal, t = −4.7, p < 0.001). We next examined regional differences in PV puncta density in the inner and outer halves of the granule cell body layer (DGCL), providing a measure of perisomatic innervation by inhibitory basket cells. We found no effect of genotype or region in PV puncta density in the cell body layer (Fig. 1C; two-way repeated measures ANOVA, genotype, F=0.712, p = 0.414; inner vs outer, F=4.181, p = 0.062; interactions, F=0.002, p = 0.966). To more directly assess perisomatic input, we determined the density of PV puncta apposed to granule cell somas, controlling for soma location. PV puncta density was significantly reduced for Pten KO cells relative to controls (Fig. 1D; two-way ANOVA with Holm-Sidak post-test, genotype, F=4.722, p = 0.041; inner vs outer, F=0.412, p = 0.527; interactions, F=0.042, p = 0.839).

PV-expressing basket cells are one of several interneuron types that innervate granule cells. To better assess overall inhibitory input, we repeated the analysis using gephyrin, a postsynaptic scaffolding protein specific to inhibitory synapses (Pizzarelli et al., 2020). Regional analyses revealed no effect of genotype, but puncta density was greater in the outer half of the layer in both controls and Pten KOs (Fig. 1E; two-way repeated measures ANOVA, genotype, F=0.287, p = 0.601; inner vs outer, F=75.402, p < 0.001; interactions, F=1.970, p = 0.184). We next examined the density of gephyrin puncta apposed to granule cell somas, controlling for soma location. Gephyrin puncta/cell surface area did not significantly differ among groups (Fig. 1F; two-way ANOVA, genotype, F=3.074, p = 0.093; inner vs outer, F=0.131, p = 0.721; interactions, F=0.008, p = 0.931). Taken together, findings are consistent with studies in the same model showing a modest reduction in frequency of spontaneous inhibitory postsynaptic currents among KO cells compared to controls (Santos et al., 2017), but an overall preservation of robust inhibitory control.

#### 3.1.1. Two-hit model to combine Pten knockout with interneuron loss

The hypothesis underlying this study is that targeted interneuron ablation would abruptly reduce inhibitory restraint of Pten KO excitatory granule cells, resulting in a rapid transition to a dramatically worsened epilepsy phenotype. To test this hypothesis, we combined loss of the mTOR negative regulator Pten from hippocampal granule cells with ablation of SST or PV interneurons. Briefly, Gli1-CreER^T2^+ /−, Pten^fl/fl^, SST-FlpO+ /− and Gli1-CreER^T2^+ /−, Pten^fl/fl^, PV-FlpO+ /− mice were treated with tamoxifen on postnatal day 21 (P21) to activate cre-recombinase and delete Pten from approximately 10% or fewer granule cells. This level of Pten loss produces hippocampal circuit abnormalities and focal seizures but not generalized convulsive seizures (LaSarge et al., 2021). Adult animals received bilateral dorsal/ventral injections of AAV9-frt-DTr targeting the dentate gyrus, allowing for Flp recombinase (FlpO)-dependent expression of the diphtheria-toxin receptor (DTr) in SST or PV interneurons. SST and PV interneurons were subsequently ablated by systemic treatment with diphtheria toxin. “2HIT” mice with Pten KO granule cells and interneuron loss were compared to controls and animals with single insults only, generating six groups: 1) Control (no KO cells or interneuron loss), 2) DGC-Pten KOs (Pten deletion from DGCs only), 3) PV ablate (PV ablation only), 4) PV 2HIT [DGC-Pten KO + PV ablate], 5) SST ablate (SST ablation only) and 6) SST 2HIT [DGC-Pten KO + SST ablate]. Full animal details are provided in Table 1.

#### 3.1.2. Impact of interneuron loss on epilepsy severity in Pten knockout mice

**Fig. 2.**
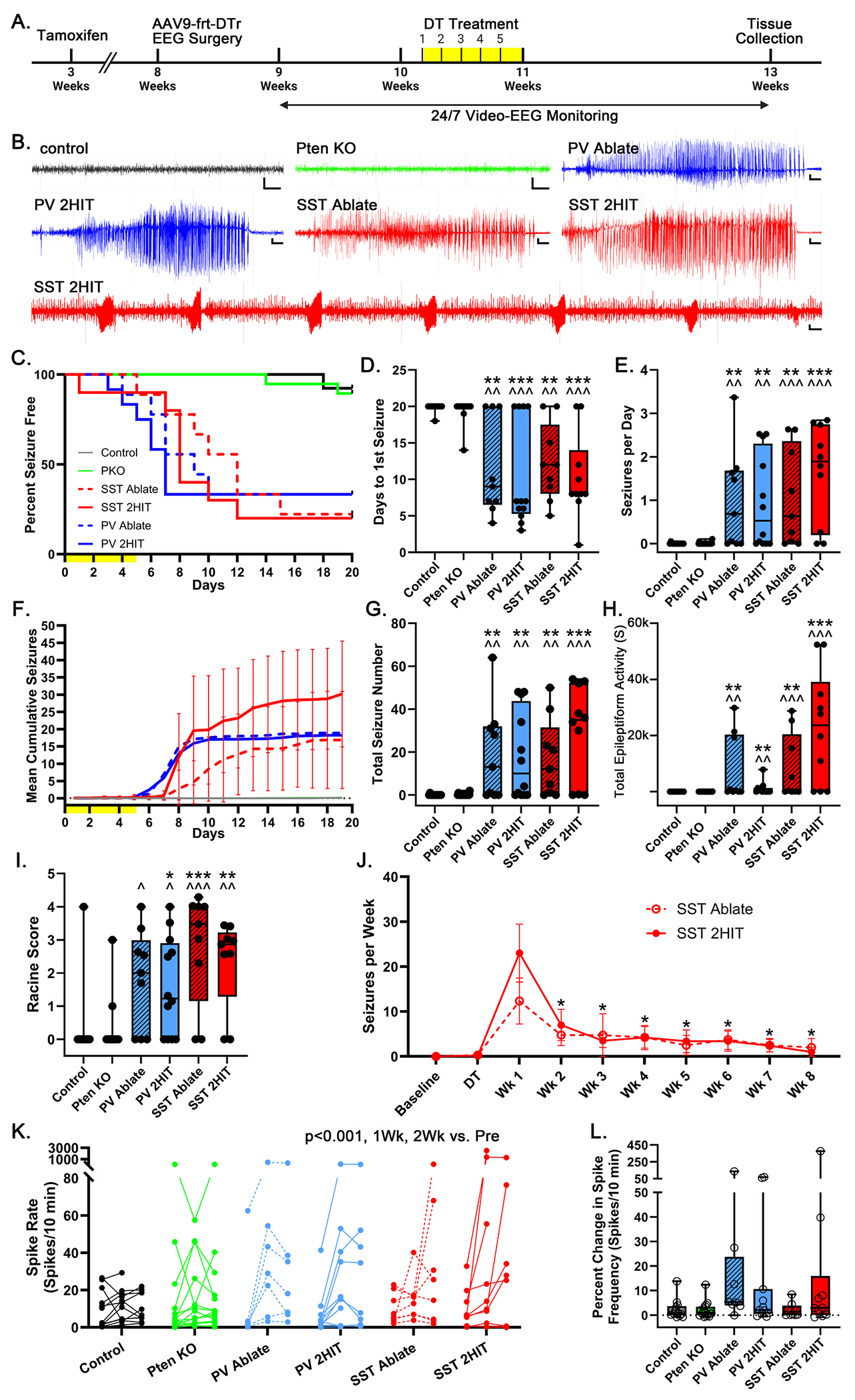
**A)** Schematic of the experimental timeline. The period of DT treatment is highlighted yellow. **B)** Example EEG traces from the 6 study groups showing normal activity, sporadic seizures, and persistent epileptiform activity. **C-D)** Kaplan-Meier curve and box plots showing the latency in days from the start of DT treatment to the first seizure. Values were set at 20 days for mice that never had seizures. **E)** Median seizure frequency during DT treatment and the following two weeks. **F-G)** Cumulative seizure frequency for each group. Most seizures in ablation groups occurred 6–10 days after the start of DT treatment, followed by infrequent seizures thereafter. 95% confidence intervals shown for SST groups in F. **H)** Total duration of epileptiform activity. **I)** Median behavioral seizure (Racine) scores for mice in each group. **J)** Seizure frequency for a subgroup of SST ablation and SST 2HIT mice monitored by 24/7 video-EEG for eight weeks after ablation. **K)** Graphs showing the rates of interictal spiking at baseline, one week after ablation, and two weeks after ablation for each mouse. **L)** Box plots show the percent increase in interictal spike frequency between baseline and post ablation. Notes: Box-plots show 10th-90th percentiles. One-way ANOVA on ranked data. *,**,***, p < 0.05, 0.01, and 0.001, respectively, vs control. ^,^^,^^^p < 0.05, 0.01, and 0.001, respectively, vs Pten KO.

Adult mice underwent 24/7 video-EEG monitoring to establish baseline seizure frequency and seizure frequency following interneuron ablation (Fig. 2A). Seizures were absent from all study animals during baseline monitoring (one SST-FlpO+/− mouse had 3 baseline seizures, likely from surgical complications, and was excluded). The majority of mice undergoing PV or SST ablation developed seizures, consistent with previous studies (Drexel et al., 2022, 2017; Spampanato and Dudek, 2017), while seizures were rare in control and DGC-Pten KO groups (Fig. 2B–C). In PV and SST ablation groups, seizure patterns ranged from sporadic to clustered, with some animals developing long periods (minutes to hours) of persistent epileptiform activity. Periods of persistent epileptiform activity were absent from controls (0/13) and Pten KOs (0/19). Persistent epileptiform activity occurred in 33% of PV ablate (3/9), 8% of PV 2HIT (1/12), 44% of SST ablate (4/9), and 70% of SST 2HIT (7/10) mice. One SST ablation and one SST 2HIT mouse died as a result of seizure activity during the two-weeks after toxin treatment. There was no mortality among other groups. Seizures typically began during the 5-day diphtheria toxin treatment period or the following week. Groups differed significantly (Fig. 2D; one way ANOVA on ranked data, F=10.4, p < 0.001) in latency to the first seizure, which was shorter for ablation groups relative to control (n = 13) and DGC-Pten KO (n = 19) groups (Holm-Sidak; PV ablate [n = 9, p = 0.004 vs control, p = 0.002 vs. DGC-Pten KO]; PV 2HIT [n = 12, p < 0.001 vs control and DGC-Pten KO], SST ablate [n = 9, p = 0.006 vs control, p = 0.004 vs. DGC-Pten KO], and SST 2HIT [n = 10, p < 0.001 vs control and DGC-Pten KO]). PV ablate, PV 2HIT, SST ablate, and SST 2HIT groups did not differ significantly from each other (p > 0.9).

Mean seizure frequency was significantly increased following PV and SST interneuron ablation (Fig. 2E; one way ANOVA on ranked data, F=10.9, p < 0.001). Animals undergoing PV or SST ablation had more seizures per day than control (n = 13) and DGC-Pten KO (n = 19) groups (Holm-Sidak; PV ablate [n = 9, p = 0.007 vs control, p = 0.006 vs. DGC-Pten KO]; PV 2HIT [n = 12, p = 0.004 vs control, p = 0.002 vs. DGC-Pten KO], SST ablate [n = 9, p = 0.001 vs control, p < 0.001 vs. DGC-Pten KO], and SST 2HIT [n = 10, p < 0.001 vs control and DGC-Pten KO]). PV ablate, PV 2HIT, SST ablate, and SST 2HIT groups did not differ significantly from each other (p > 0.6). To assess statistical power, we conducted *t*-tests on seizure frequency comparing ablation-only and 2HIT groups. Groups did not differ significantly (PV ablate vs. PV 2HIT, t=−0.07, 19df, p = 0.946; SST ablate vs. SST 2HIT, t = −1.09, 17df, p = 0.292). Power to detect a doubling and tripling in seizure frequency was 0.469 and 0.964 for PV groups, respectively, and 0.537 and 0.984 for SST groups.

Most seizures tended to occur in the first week after PV and SST ablation, followed by a relative plateauing (Fig. 2F). Total seizure number over the DT-treatment and two-week post-treatment periods was significantly increased for ablation groups relative to control (n = 13) and DGC-Pten KO (n = 19) groups (Fig. 2G; one way ANOVA on ranked data, F=10.8, p < 0.001; Holm-Sidak; PV ablate [n = 9, p = 0.006 vs control, p = 0.005 vs. DGC-Pten KO]; PV 2HIT [n = 12, p = 0.003 vs control, p = 0.002 vs. DGC-Pten KO], SST ablate [n = 9, p = 0.002 vs control and DGC-Pten KO], and SST 2HIT [n = 10, p < 0.001 vs control and DGC-Pten KO]). PV ablate, PV 2HIT, SST ablate, and SST 2HIT groups did not differ significantly from each other (p > 0.9).

To account for the development of persistent epileptiform activity in some mice, we also calculated the total duration of seizure and seizure-like (epileptiform) activity over the DT and post-treatment periods. The total duration of epileptiform activity was significantly increased among PV and SST ablation groups relative to control (n = 13) and DGC-Pten KO (n = 19) groups (Fig. 2H; one way ANOVA on ranked data, F=11.5, p < 0.001; Holm-Sidak; PV ablate [n = 9, p = 0.006 vs control, p = 0.004 vs. DGC-Pten KO]; PV 2HIT [n = 12, p = 0.009 vs control, p = 0.007 vs. DGC-Pten KO], SST ablate [n = 9, p = 0.001 vs control, p < 0.001 vs. DGC-Pten KO] and SST 2HIT [n = 10, p < 0.001 vs control and DGC-Pten KO]). PV ablate, PV 2HIT, SST ablate, and SST 2HIT groups did not differ significantly from each other (p > 0.1).

Electrographic seizures were associated with typical seizure behaviors, which were characterized using a modified 5-point Racine scale for behavioral seizure scoring (Racine, 1972). Median behavioral seizure scores were significantly higher, or trended higher, among PV and SST ablation groups relative to control (n = 13) and DGC-Pten KO (n = 19) groups (Fig. 2I; one way ANOVA on ranked data, F=8.6, p < 0.001; Holm-Sidak; PV ablate [n = 9, p = 0.052 vs control, p = 0.028 vs. DGC-Pten KO]; PV 2HIT [n = 12, p = 0.046 vs control, p = 0.021 vs. DGC-Pten KO], SST ablate [n = 9, p < 0.001 vs control and DGC-Pten KO], and SST 2HIT [n = 9, p = 0.006 vs control, p < 0.002 vs. DGC-Pten KO]). PV ablate, PV 2HIT, SST ablate, and SST 2HIT groups did not differ significantly from each other (p > 0.4).

Epilepsy severity among SST ablation groups showed a trend towards higher values across several measures in the two-week period after DT treatment (Fig. 2, F, H, I). To query whether an effect of genotype might develop over time, 24/7 video-EEG recording was continued for an additional 6 weeks for a subset of mice (SST ablate, n = 4; SST 2HIT, n = 5). There was no mortality among the SST ablation group, while one SST 2HIT mouse died during seizures in the 8th week after interneuron ablation. Analysis of seizure count per week for all SST ablation and SST 2HIT mice across the full recording period revealed a significant effect for timepoint, but not for Pten deletion (Fig. 2J; two-way ANOVA on square root transformed data, timepoint, p < 0.001, SST ablation vs SST 2HIT, p = 0.696). Seizure frequency significantly decreased for both SST ablate and SST 2HIT groups during the 2–8 week extended monitoring period after ablation compared to week one (Holm-Sidak, p < 0.05 for all comparisons). Findings indicate that the presence of Pten KO cells does not impact seizure frequency in the months after SST interneuron ablation.

As an additional measure of epileptogenesis, we assessed the frequency of interictal spikes. Spike frequency was determined from EEG data on the day before the start of DT treatment, and on days 7 and 14 after the last DT dose. A subset of animals undergoing ablation exhibited dramatic increases in spike frequency following ablation, particularly in SST groups (Fig. 2K). Statistical analyses revealed a main effect of time (two-way repeated measures ANOVA, F=32.7, p < 0.001) but no effect of genotype (F=0.6, p = 0.703) or interactions (F=1.8, p = 0.069). Spike frequency 7 and 14 days after DT treatment was significantly increased relative to the day before treatment (Bonferroni *t*-test, p < 0.001). Normalizing the data to compare changes in spike frequency over baseline did not reveal a significant difference among groups (Fig. 2L; one way ANOVA on ranked data, F=2.0, p = 0.096).

#### 3.1.3. Efficacy of Pten deletion and interneuron ablation

Tamoxifen-inducible cre-mediated deletion of Pten from granule cells and AAV flpO-mediated expression of the diphtheria toxin receptor in interneurons can vary among animals depending on a variety of factors, including tamoxifen absorption and surgical targeting. To account for this variability and incorporate it into the statistical approach, histological studies were conducted to quantify Pten deletion rates and interneuron densities.

**Fig. 3.**
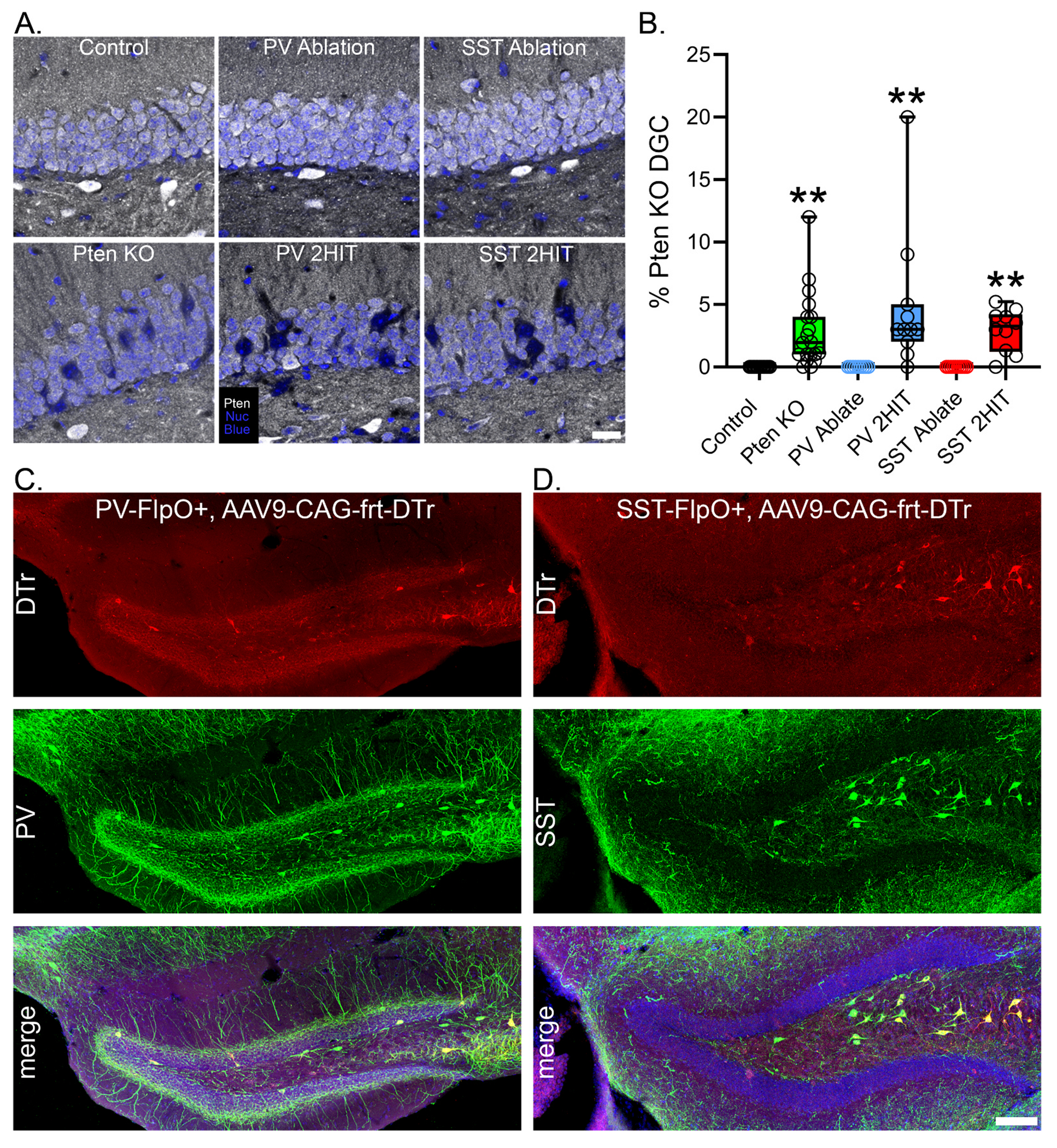
**A)** Confocal images of the dentate granule cell layer for each group. Tissue was immunostained for Pten and counterstained with Nuclear Blue. Pten KO DGCs are identified by the absence of Pten staining, appearing as darker blue cells in the images. Scale = 20 μm. **B)** Percentage of dentate granule cells which were Pten immunonegative (Pten KO) in each group. **, p < 0.01 vs control, PV ablate and SST ablate. **C)** Immunostaining for diphtheria toxin receptor (DTr) and PV in a PV-FlpO positive mouse injected with AAV9-CAG-frt-DTr. Immunostaining shows double labeling of PV interneurons in the hilus. **D)** Immunostaining for DTr and SST in a SST-FlpO positive mouse injected with AAV9-CAG-frt-DTr. Immunostaining shows double labeling of SST interneurons in the hilus. Scale = 100 μm.

All mice were immunostained for Pten to establish the percentage of KO granule cells (Fig. 3A). In control, PV ablate, and SST ablate groups, all granule cells were immunoreactive for Pten. Conversely, Pten immunostaining was absent from a median of 2.0% [quartiles 1–4] of granule cells from Pten KO mice, 3.0% [2–5] from PV 2HIT mice and 3.3% [1–4] from SST 2HIT mice. Statistical analyses revealed a main effect of group (Fig. 3B; Kruskal-Wallis, H=48.2, df=5, p < 0.001), with DGC-Pten KO, PV 2HIT, and SST 2HIT groups having significantly more KO cells than control, PV ablate, and SST ablate groups (Dunn’s, p < 0.01) but not differing from each other (Dunn’s, p = 1). Findings demonstrate similar Pten deletion rates among Pten KO and 2HIT groups.

Saline-treated controls (no toxin exposure) were used to evaluate diphtheria toxin receptor expression in PV and SST interneurons. Immunostaining for the diphtheria toxin receptor colocalized with cells immunoreactive for PV (Fig. 3C) and SST (Fig. 3D), with no evidence of significant receptor expression outside of the targeted interneuron types. Findings confirm the efficacy of targeting dentate interneurons with the PV-FlpO and SST-FlpO mouse lines.

**Fig. 4.**
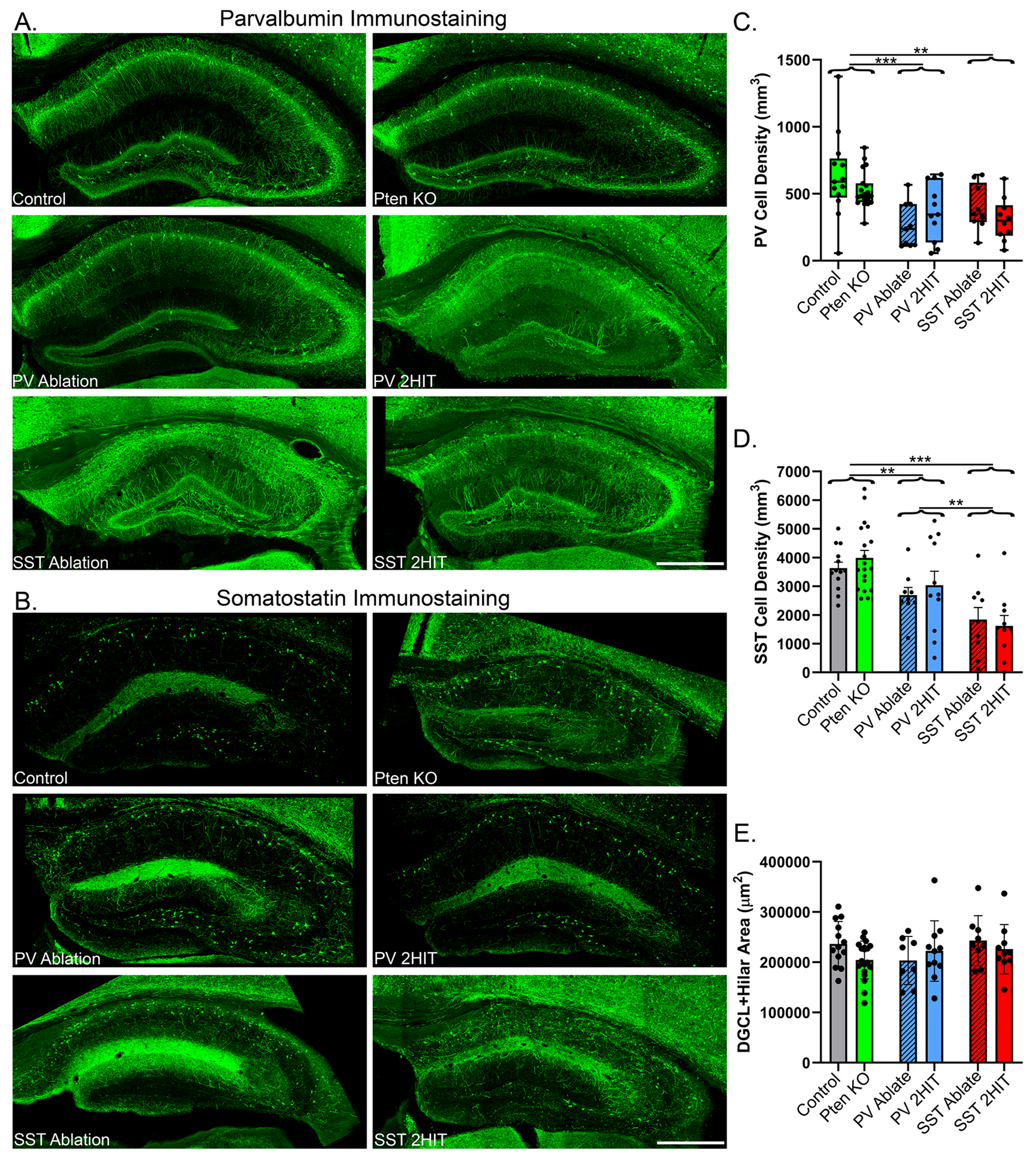
**A)** Representative images of hippocampal PV immunostaining from animals in the six experimental groups. **B)** Representative images of hippocampal SST immunostaining from animals in the six experimental groups. Scale for A and B = 500 μm. **C)** Box plot showing the density of PV immunoreactive cells in the dentate hilus for each group. **D)** Bar graph showing the density of SST immunoreactive cells in the dentate hilus for each group. **E)** Bar graph showing the area of the dentate hilus for each group. *p < 0.05, **p < 0.01, ***p < 0.001. Bars represent animal means ± SEM.

To quantify interneuron loss, all mice were immunostained for PV (Fig. 4A) and SST (Fig. 4B), followed by bilateral cell counts of serial sections collected through the dorsal-ventral axis of the hippocampal dentate gyrus. Quantification of PV cell density showed significant reductions following PV interneuron ablation relative to no ablation groups (Fig. 4C; Two-way ANOVA on square root transformed data, main effect of treatment (ablation/no ablation; F=12.5, p < 0.001) with no effect of genotype (wildtype/KO; F=0.1, p = 0.708) or interactions (p = 0.247). Holm-Sidak post-tests within treatment demonstrated significant differences for PV ablation versus no ablation groups (p < 0.001) and SST ablation versus no ablation groups (p = 0.001). PV ablation and SST ablation groups, however, did not differ from each other (p = 0.410). SST cell density was also significantly reduced following SST interneuron ablation (Fig. 4D; Two-way ANOVA, main effect of treatment (ablation/no ablation; F=18.9, p < 0.001) with no effect of genotype (wildtype/KO; F=0.3, p = 0.578) or interactions (p = 0.659). Holm-Sidak post-tests within treatment demonstrated significant differences for PV ablation groups versus no ablation groups (p < 0.007) and SST ablation groups versus no ablation (p < 0.001). SST cell density was also significantly lower in SST ablation vs PV ablation groups (p = 0.007). Findings demonstrate that PV and SST ablation are effective, but also suggest some overlap in interneuron targeting with the PV-FlpO and SST-FlpO lines. PV cell density was similarly reduced in PV and SST ablation groups, while SST cell density in PV ablation groups was intermediate between SST ablation and control. Subsets of interneurons are known to co-express PV and SST (Jinno and Kosaka, 2000), so some overlap is expected. In addition, seizures can induce interneuron death (Klein et al., 2025), which could also contribute to loss of PV cells in SST ablated mice. Stated plainly, SST ablation can be viewed as the more severe of the two treatments, eliminating the same number of PV interneurons as PV ablation, but eliminating more SST neurons.

The area of the dentate gyrus has been observed to change in several epilepsy models (Bouilleret et al., 1999; Gröticke et al., 2007; Murphy et al., 2012), potentially reflecting effects of cell loss, neurogenesis and/or neuronal hypertrophy. We evaluated, therefore, whether the area of the dentate might vary among groups. For the present study, dentate area was found to be statistically similar among groups (Fig. 4E, Two-way ANOVA with genotype [F=1.4, p = 0.243] and treatment [F=0.3, p = 0.740] as factors). A significant interaction was not observed (F=2.1, p = 0.128).

#### 3.1.4. Relationship between DGC-Pten knockout load, interneuron loss and epilepsy severity

**Fig. 5.**
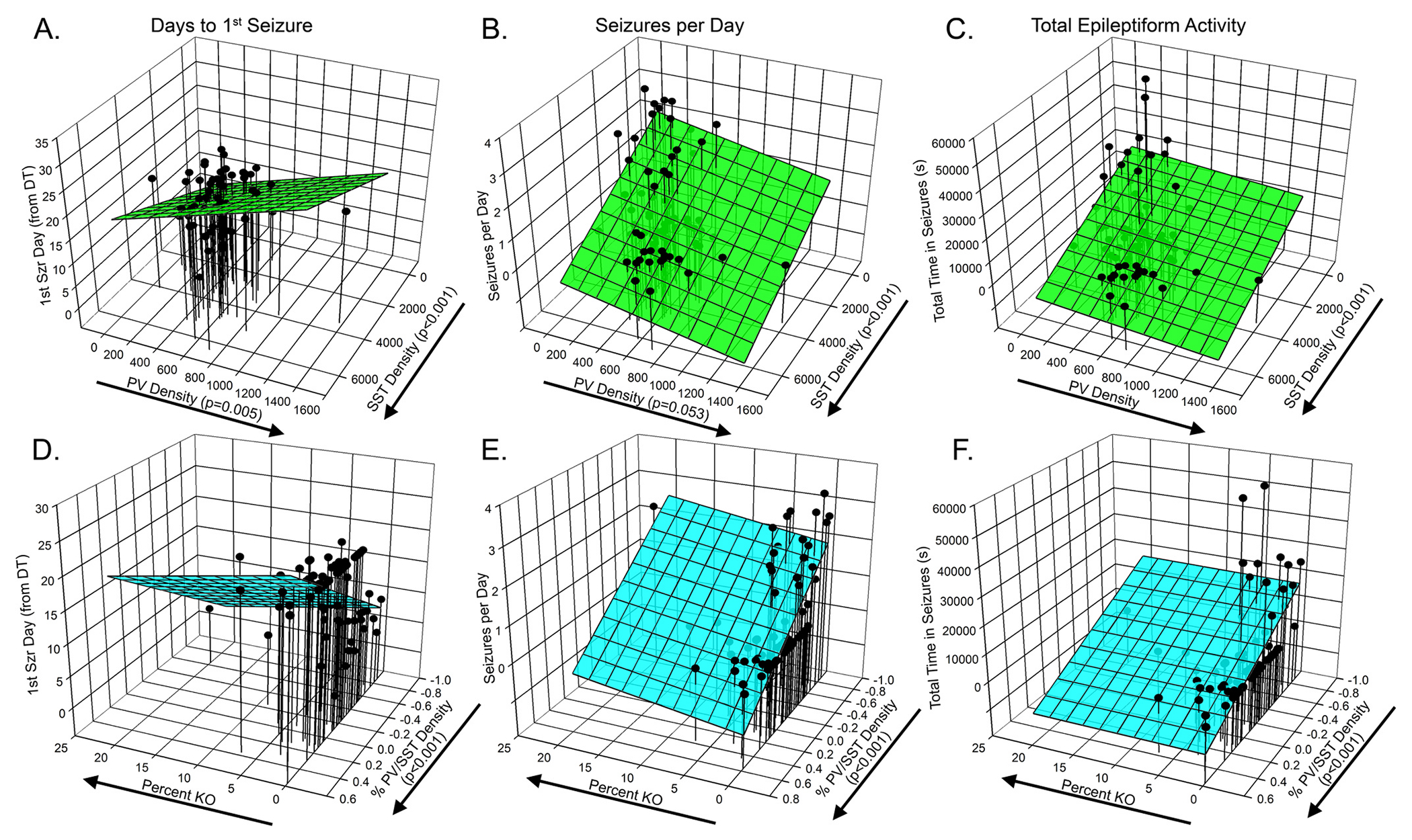
Three-dimensional graphs from multiple linear regression analyses examining the relationships among measures of epilepsy severity, the percentage of Pten KO cells, hilar PV interneuron density, and hilar SST interneuron density. **A-C)** Epilepsy measures (y axis) plotted against PV (x axis) and SST (z axis) density. The percentage of Pten KO cells is not shown. **D-F)** Epilepsy measures (y axis) plotted against the percentage of Pten KO granule cells (x axis) and a combined metric of PV and SST cell density (z axis). Arrows note the direction of increasing percentage of KO cells or interneuron density.

To elucidate which variables – Pten KO load, PV density, or SST density – contributed most to observed seizure phenotypes, multiple linear regression analyses were conducted. Analyses indicate that seizure latency (Fig. 5A) can be predicted by PV density (p = 0.005) and SST density (p < 0.001) but not by Pten KO cell load (p = 0.243). Similarly, seizure frequency (Fig. 5B) showed a trend towards being predicted by PV density (p = 0.053) and was predicted by SST density (p < 0.001) but not by Pten KO cell load (p = 0.292). Total time spent in epileptiform activity (Fig. 5C), on the other hand, was predicted by SST density (p < 0.001), but not by PV density (p = 0.439) or KO cell load (p = 0.945). To further query the data set and help with data visualization, PV and SST cell density scores were normalized and combined to calculate the percentage of interneuron loss for each mouse (Fig. 5D–F). Regression analyses produced a similar result, with seizure latency (Fig. 5D, %loss, p < 0.001; KO load, p = 0.222), frequency (Fig. 5E, % loss, p < 0.001; KO load, p = 0.236), and total duration of epileptiform activity (Fig. 5F, %loss, p < 0.001; KO load, p = 0.941) being driven by interneuron loss but not Pten KO cell density.

#### 3.1.5. SST interneuron sprouting

**Fig. 6.**
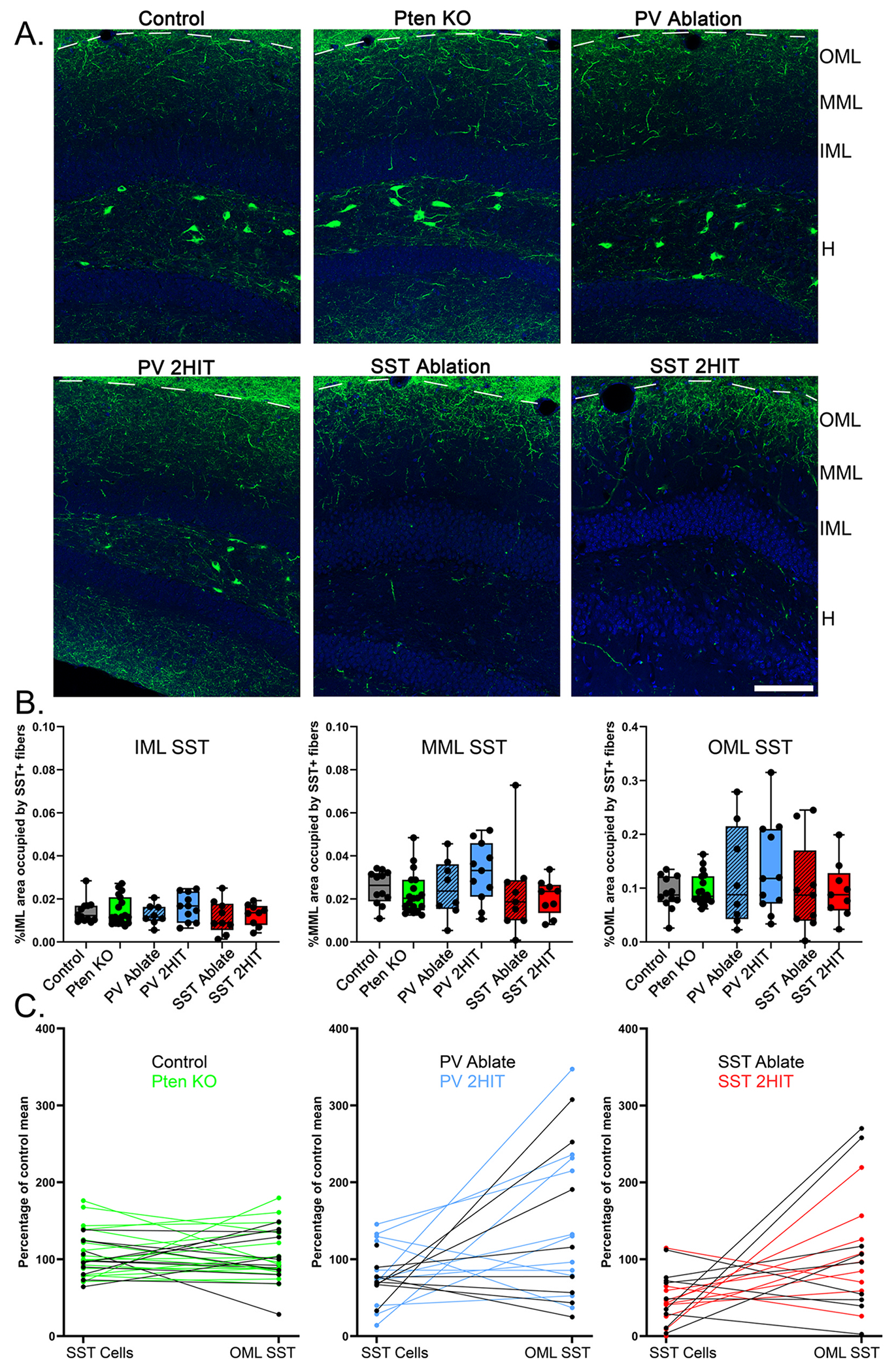
**A)** Confocal images show SST immunostaining in the dentate gyri of mice from each experimental group. SST immunoreactive fibers were prominent in the dentate outer molecular later (OML), but occasionally extended into the middle (MML) and inner (IML) molecular layers. SST immunoreactive somas are evident in the hilar (H) region of all groups except SST ablation. **B)** Box plots show the percentage of inner, middle and outer molecular layers occupied by SST immunoreactive fibers. Despite ablation of hilar SST interneurons in PV and SST groups, fiber density did not differ significantly. **C)** Line graphs for each study animal depict the density of SST cells in the dentate hilus (SST cells), normalized as a percentage of control, to the area of the outer molecular layer occupied by SST+ fibers (OML SST), normalized as a percentage of control. Control and Pten KO animals fall around 100%, indicating little deviation from the control mean. PV and SST ablation animals often show reduced hilar cell density (<100%) but preserved or increased fiber density in the OML (>100%).

An intriguing finding of the present study is that interneuron ablation produced a transient cluster of seizures, followed by a significant reduction in seizure incidence over the course of weeks. Sprouting of surviving interneurons could contribute to a restoration of inhibitory input to granule cells. To explore whether this might be the case, we measured the area of the dentate inner (IML), middle (MML) and outer (OML) molecular layers occupied by SST-immunoreactive fibers. Despite significant reductions in the density of hilar SST neurons (Fig. 4D), the percentage of the three layers occupied by SST fibers was similar among groups (Fig. 6A–B; Kruskal-Wallis; IML, H=2.8, p = 0.735; MML, H=6.7, p = 0.240; OML, H=1.4, p = 0.928). To better visualize the relationship between cell density and fiber area in each animal, the two values for each mouse were normalized as a percentage of the control mean. Normalized values for control and Pten KO mice were around 100% of the control mean, whereas many animals in ablation groups had fewer hilar SST cells than controls (<100%), but a greater density of SST fibers in the OML (Fig. 6C). Findings may reflect sprouting of SST cells in the CA1 region into the dentate gyrus, as has been described for other epilepsy models (Peng et al., 2013).

## 4. Discussion

In this study, we tested a two-hit model of epileptogenesis in mTORopathies, in which small numbers of mTOR hyperactive (Pten KO) excitatory granule cells were introduced during development, followed by ablation of PV and SST interneurons in adulthood. PV and SST interneurons are critical regulators of granule cell activity. Pten KO cells were present at the target frequency in study mice, and ablation effectively reduced PV and SST cell density to levels equal to or exceeding the loss evident in mTORopathy models (Yang et al., 2022; LaSarge et al., 2021; Drake et al., 2024; Zhong et al., 2021) and patients (Nakagawa et al., 2017; Thom et al., 2003; Cheng et al., 2022; Zamecnik et al., 2006). These aspects of mTORopathies, therefore, were effectively modeled. Ablation of either PV or SST interneurons in the dentate gyrus produced clusters of seizures during the first week, followed by sporadic seizures in subsequent weeks. In contrast to our hypothesis, however, the presence of Pten KO cells did not significantly impact seizure severity. Interneuron ablation produced similar seizure phenotypes in mice with and without KO cells. Findings support the potential importance of interneuron loss – and the resulting impairment in excitatory/inhibitory balance – as a key driver of epileptogenesis. The absence of an interaction between interneuron loss and Pten KO cells, however, implies that small numbers of KO cells are insufficient to drive seizures even when disinhibited.

### 4.1. Rationale and limitations of the experimental approach

We proposed that interneuron loss would disinhibit Pten KO granule cells, which would drive a dramatic worsening of seizure phenotypes. The hypothesis and approach are based on several observations. To start, Pten KO cells must be the proximal cause of epilepsy, at least in animal models in which the disease-causing manipulation (Pten deletion) is known and tightly controlled (Pun et al., 2012; Yonan et al., 2024). Further, Pten KO granule cells develop features consistent with increased excitability (Santos et al., 2017; Luikart et al., 2011; Skelton et al., 2019; Williams et al., 2015; Haws et al., 2014) and are therefore an appealing candidate for driving seizures. Pten KOs cells also possess many features of hub cells (Kwon et al., 2006; LaSarge et al., 2015; Yonan and Steward, 2023), a highly connected cell class hypothesized to destabilize networks and initiate seizures (Bui et al., 2015). As the name implies, hub cells possess large numbers of inputs and outputs and are thus positioned to produce outsized impacts on connected networks. Computer modeling studies have found that designating just 5% of granule cells as hub cells is sufficient to produce seizures (Morgan and Soltesz, 2008). The number aligns well with our experimental observations that focal seizures appear in the DGC-Pten KO model at a threshold of around 3–5%, while generalized seizures appear at a threshold of about 10% (LaSarge et al., 2021; Pun et al., 2012). A limitation of the cortical EEG recordings used here is that they only reliably detect generalized seizures, so changes in focal seizures may not be captured.

Studies take advantage of the unique inhibitory circuitry of the dentate gyrus. The structure contains a spatially-constrained population of PV and SST interneurons in the dentate hilus which have, as their primary target, the somas and distal dendrites of dentate granule cells (Houser, 2007). PV and SST neurons play similar roles in cortex, innervating somas and distal dendrites of cortical pyramidal cells, respectively (Llorca and Deogracias, 2022). However, cortical interneurons show tremendous diversity in structure, function and laminar position (Sultan and Shi, 2018), making it more challenging to target a defined interneuron subtype innervating a specific class of excitatory neuron. The anatomy of the dentate gyrus, therefore, provides an ideal opportunity to selectively manipulate critical interneuron populations regulating mTOR hyperactive neurons. That said, it should be noted that differences in circuitry could also produce different results in hippocampus vs. cortex. Further studies will be needed to establish the impact of interneuron loss in cortical mTORopathy models.

#### 4.1.1. Role of interneuron loss in mTORopathies

Findings strongly suggest that our two-hit model is incorrect. Several scenarios can be considered to explain this result. Firstly, it is possible that ablation of either PV or SST cells does not sufficiently disinhibit Pten KO cells. Surviving interneurons might be able to maintain stable levels of inhibitory input to KO cells, perhaps by increasing their connectivity or firing rates (Cepeda et al., 2012; Halabisky et al., 2010; Hunt et al., 2011). We did observe an apparent recovery of SST immunoreactive fibers in the dentate gyrus, likely mediated by sprouting of SST+ OLM cells in CA1, as has been described in other epilepsy models (Peng et al., 2013; Zhang et al., 2009). Sprouting takes time to develop, however, and thus would seem unlikely to be able to restore inhibition in hours or even days. Pten KO cells might also be kept in check by other interneuron types innervating the dentate (Houser, 2007; Tzilivaki et al., 2023), an interpretation consistent with hippocampal slice physiology data indicating a limited role for PV interneurons in basal synaptic inhibition of granule cells (Afrasiabi et al., 2022). Alternatively, tonic inhibition mediated by extrasynaptic GABA receptors could play the predominant role rather than phasic input from PV and SST cells (Sperk et al., 2021; Li et al., 2013; Walker and Kullmann, 2012). The complexity of the model prevented us from assessing single cell physiology or circuit abnormalities in brain slices from the animals (LaSarge et al., 2016), so we cannot exclude these possibilities. A second possibility is that disinhibited Pten KO cells – at the percentages tested in the present study – are insufficient to drive seizures regardless of their level of inhibitory restraint. We intentionally examined subthreshold KO cell loads to test our two-hit model, but perhaps small numbers of KO cells, even when disinhibited, have minimal impact on seizure incidence. Increasing KO cell loads above 10% – the level at which spontaneous seizures begin to appear – might produce different results. That said, two PV 2HIT mice with KO rates of 9 and 20% had seizure rates similar to animals with lower KO cell loads, favoring other interpretations. A third possibility is that KO cells and interneuron loss act to produce seizures by distinct, non-overlapping mechanisms. In this scenario, a subthreshold load of KO cells remains subthreshold even with interneuron loss, and no synergistic effects would be expected. Lastly, mTOR mutant neurons may only initiate the process of epileptogenesis by provoking interneuron loss. Once interneuron loss has occurred, the broader granule cell network becomes disinhibited, and seizure activity is driven by the larger population of wildtype cells. In this model, mTOR hyperactive neurons begin the disease process, but do not directly drive seizures. This latter model is consistent with some studies implicating non-mutant cells surrounding the lesion, rather than mutant cells within the lesion core, as playing a greater role in driving seizures (Koh et al., 2021; Almacellas Barbanoj et al., 2024). The model is also consistent with single cell calcium imaging of epileptogenesis in organotypic hippocampal slices, in which seizures initiation appears to occur at random from cells within the circuit rather than recurrently from a single or small group of cells (Lau et al., 2022). On the other hand, recent work using senolytic drugs in a mouse model to ablate mTOR hyperactive cortical neurons, which acquire a senescent phenotype making them vulnerable to the drugs, demonstrated a significant reduction in seizure frequency (Ribierre et al., 2024). Findings suggest that mTOR hyperactive cortical neurons are critical for driving seizures in this model. To discriminate among these hypotheses, additional studies are needed to assess whether the role played by mTOR hyperactive neurons varies by gene target, mutant cell load or affected brain region (e.g. hippocampus vs cortex).

#### 4.1.2. Pten knockout granule cell-mediated mechanisms of epileptogenesis

While findings do not support our two-hit model, Pten KO granule cells are still the proximal cause of epilepsy in the mice. How then might these abnormal neurons initiate epileptogenesis? Secondary interneuron loss remains an appealing possibility. Pten KO cells generate focal seizures, which could progressively damage local circuit interneurons. Our prior work in the model indicates that focal seizures and circuit abnormalities are present in mice with KO cell loads of 3–5% (LaSarge et al., 2021), so this mechanism seems plausible. Alternatively, mTOR mutant neurons could drive interneuron loss via non-synaptic mechanisms. mTOR hyperactivation leads to the overproduction of many proteins, including growth factors (Guo et al., 2024; Brugarolas et al., 2003) which could dysregulate interneuron migration, differentiation or survival. mTOR hyperactive neurons can also acquire a senescence associated secretory phenotype (SASP), releasing cytokines which might have toxic effects on interneurons (Ribierre et al., 2024). Findings don’t rule out the possibility, however, that interneuron loss observed in mTORopathy models (Yang et al., 2022; LaSarge et al., 2021; Drake et al., 2024; Zhong et al., 2021) and patients (Nakagawa et al., 2017; Thom et al., 2003; Cheng et al., 2022; Zamecnik et al., 2006) is an epiphenomenon. Generalized seizures could be driven directly by synaptic output from mutant cells, but only when present in sufficient numbers. Seizures could also be driven by effects of KO neurons on non-neuronal cells, for example, through cytokine release, inflammation, or blood-brain barrier disruption (Dusing et al., 2023; van Vliet et al., 2012; Frigerio et al., 2012). While many questions are left unanswered, powerful combinations of new models, transgenic mice, and viral transfection tools now make it possible to conduct precise interrogations of epileptic circuits. Futures studies using these tools will provide critical insights.

## 5. Conclusions

Interneuron loss is a characteristic feature of a wide range of epilepsies, including mTORopathies. Here, we tested the hypothesis that epileptogenesis in the mTORopathies proceeds by a two-hit model, wherein mTOR hyperactive neurons promote interneuron loss, which in turn further disinhibits the hyperactive neurons and drives seizures. Rather than supporting this hypothesis, we found no evidence that abrupt loss of hippocampal interneurons increases the ictogenic potential of Pten KO granule cells. While dramatic increases in seizure incidence following interneuron ablation alone further establish the importance of inhibitory cells in maintaining excitatory/inhibitory balance, the absence of an interaction with Pten KO cells indicates there is still much to be learned about the function of pathological hippocampal circuits in epilepsy.

## Supplementary Material

Supplemental Table 1

Supplemental Table 2

## Figures and Tables

**Table 1:** Study Group Details.

Group	Genotype	Treatment	Number
Control	Gli1-CreER^T2^ (−/−), Pten^fl/fl^, SST-FlpO (+/−)	Saline	4 (2 F, 2 M)
	Gli1-CreER^T2^ (−/−), Pten^fl/fl^, PV-FlpO (−/−)	Saline	2 (1 F, 1 M)
	Gli1-CreER^T2^ (−/−), Pten^fl/fl^, PV-FlpO (+/−)	Saline	7 (2 F, 5 M)
DGC-Pten KO	Gli1-CreER^T2^ (+/−), Pten^fl/fl^	Saline	14 (4 F, 10 M)
	Gli1-CreER^T2^ (+/−), Pten^fl/fl^	DT	5 (3 F, 2 M)
SST Ablate	Gli1-CreER^T2^ (−/−), Pten^fl/fl^, SST-FlpO (+/−)	DT	9 (1 F, 8 M)
SST 2HIT (DGC-Pten KO + SST Ablate)	Gli1-CreER^T2^ (+/−), Pten^fl/fl^, SST-FlpO (+/−)	DT	10 (2 F, 8 M)
PV Ablate	Gli1-CreER^T2^ (−/−), Pten^wt/wt^, PV-FlpO (+/−)	DT	2 (2 M)
	Gli1-CreER^T2^ (−/−), Pten^fl/fl^, PV-FlpO (+/−)	DT	7 (4 F, 3 M)
PV 2HIT (DGC-Pten KO + PV Ablate)	Gli1-CreER^T2^ (+/−), Pten^fl/fl^, PV-FlpO (+/−)	DT	12 (5 F, 7 M)

## Appendix A. Supporting information

Supplementary data associated with this article can be found in the online version at doi:10.1016/j.pneurobio.2026.102925.
