# Supplementary material for: Targeted interneuron ablation in an mTORopathy model: Testing a two-hit mechanism of epileptogenesis
Source: Prog Neurobiol. Author manuscript; Available in PMC 2026 Jul 14. (PMC13366186; doi:10.1016/j.pneurobio.2026.102925)
Supplement: Supplemental Table 1 [file NIHMS2190132-supplement-Supplemental_Table_1.docx]

| **Supplemental Table 1: Key Study Resources** | |
| --- | --- |
| **Resources** | **RRID** |
| Gli1-CreER^T2^ Transgenic Mouse | IMSR_JAX:007913 |
| Pten Transgenic Mouse | IMSR_JAX:006440 |
| SST-FlpO Transgenic Mouse | IMSR_JAX:028579 |
| PV-FlpO Transgenic Mouse | IMSR_JAX:022730 |
| tdTomato Reporter Transgenic Mouse | IMSR_JAX:007914 |
| Allen Mouse Brain Reference Atlas | SCR_002978 |
| Nikon A1R Confocal Laser Scanning Microscope | SCR_020317 |
| NIS-Elements AR (Version 5.42.03) | SCR_014329 |
| Cincinnati Children’s Bio-Imaging and Analysis Facility | SCR_022628 |
| Imaris (Version 10.1.1) | SCR_007370 |
| Neurolucida 360 | SCR_016788 |
| Neurolucida Explorer | SCR_017348 |
| SigmaPlot (Version 15.0) | SCR_003210 |
| GraphPad Prism (Version 10) | SCR_002798 |
