# Supplementary material for: Targeted interneuron ablation in an mTORopathy model: Testing a two-hit mechanism of epileptogenesis
Source: Prog Neurobiol. Author manuscript; Available in PMC 2026 Jul 14. (PMC13366186; doi:10.1016/j.pneurobio.2026.102925)
Supplement: Supplemental Table 2 [file NIHMS2190132-supplement-Supplemental_Table_2.docx]

| **Supplemental Table 2: Primary and Secondary Antibodies** | | | | |
| --- | --- | --- | --- | --- |
| **Antibody** | **Target** | **Species** | **Concentration** | **RRID** |
| **Primaries** | DTr (HB-EGF) | Goat | 1:500 | AB_354429 |
|  | Somatostatin | Rabbit | 1:200 | AB_2789834 |
|  | Parvalbumin | Guinea Pig | 1:1000 | AB_2156476 |
|  | Pten | Rabbit | 1:250 | AB_390810 |
|  | Gephyrin | Chicken | 1:1000 | AB_2943527 |
| **Secondaries** | DyLight™ 405 AffiniPure Donkey Anti-Guinea Pig IgG (H+L) | Donkey | 1:750 | AB_2340470 |
|  | Alexa Fluor 488 anti-rabbit IgG (H+L) | Donkey | 1:750 | AB_2535792 |
|  | Alexa Fluor 568 anti-chicken IgG (H+L) | Donkey | 1:750 | AB_2921072 |
|  | Alexa Fluor 568 anti-goat IgG (H+L) | Donkey | 1:750 | AB_2534104 |
|  | Alexa Fluor 647 anti-rabbit IgG (H+L) | Donkey | 1:750 | AB_2536183 |
|  | Alexa Fluor 647 AffiniPure anti-guinea pig IgG (H+L) | Donkey | 1:750 | AB_2340476 |
